# Cullin 3/KCTD5 Promotes the Ubiqutination of Rho Guanine Nucleotide Dissociation Inhibitor 1 and Regulates Its Stability

**DOI:** 10.4014/jmb.2007.07033

**Published:** 2020-08-28

**Authors:** Hee Jun Cho, Ki-Jun Ryu, Kyoung Eun Baek, Jeewon Lim, Taeyoung Kim, Chae Yeong Song, Jiyun Yoo, Hee Gu Lee

**Affiliations:** 1 Immunotherapy Research Center, Korea Research Institute of Bioscience and Biotechnology (KRIBB), Daejeon 34141, Republic of Korea; 2 Division of Applied Life Science, Research Institute of Life Sciences, Gyeongsang National University, Jinju 52828, Republic of Korea

**Keywords:** RhoGDI1, Rho GTPases, Cullin3, KCTD5, ubiquitination

## Abstract

Rho guanine nucleotide dissociation inhibitor 1 (RhoGDI1) plays important roles in numerous cellular processes, including cell motility, adhesion, and proliferation, by regulating the activity of Rho GTPases. Its expression is altered in various human cancers and is associated with malignant progression. Here, we show that RhoGDI1 interacts with Cullin 3 (CUL3), a scaffold protein for E3 ubiquitin ligase complexes. Ectopic expression of CUL3 increases the ubiquitination of RhoGDI1. Furthermore, potassium channel tetramerization domain containing 5 (KCTD5) also binds to RhoGDI1 and increases its interaction with CUL3. Ectopic expression of KCTD5 increases the ubiquitination of RhoGDI1, whereas its knockdown by RNA interference has the opposite effect. Depletion of KCTD5 or expression of dominant-negative CUL3 (DN-CUL3) enhances the stability of RhoGDI1. Our findings reveal a previously unknown mechanism for controlling RhoGDI1 degradation that involves a CUL3/KCTD5 ubiquitin ligase complex.

## Introduction

Ubiquitination is a post-translational modification involving the covalent attachment of a ubiquitin moiety to a lysine residue and is known to regulate the localization, activity, and stability of the substrate proteins [1, 2]. There are two types of E3 ubiquitin ligases, namely, the RING (really interesting new gene) and HECT (homologous to the E6AP carboxyl terminus) families [3]. Cullins are a family of hydrophobic scaffold proteins that provide support for ubiquitin ligases. They share a homologous C-terminus that binds to the RING-box protein (Rbx1), but diverge at the N-terminal substrate recruiting domain [4, 5]. Members of a subclass of the RING protein family interact with Cullins to create the aptly-named Cullin-RING ligases (CRLs), whose ubiquitin ligase activities are involved in many biological processes. Cullin 3-RING ligases (CRL3s) typically consist of the scaffold protein Cullin 3 (CUL3), Rbx1, and a Bric-a-brac/Tramtrack/Broad (BTB) protein. These component proteins collaborate in the ubiquitination of substrate proteins [6-8].

Rho GTPases are involved in various cellular processes, including cell motility, adhesion, and proliferation [9]. They act as molecular switches, cycling between an inactive guanosine diphosphate (GDP)-bound form in the cytoplasm and an active guanosine triphosphate (GTP)-bound form in the membrane [10]. This cycling is tightly regulated by Rho guanine nucleotide exchange factors (RhoGEFs), *i.e.*, proteins that promote the exchange of GDP for GTP, and Rho GTPase-activating proteins (RhoGAPs), which catalyze intrinsic GTP hydrolysis, thereby inactivating Rho GTPases [11, 12]. Rho guanine nucleotide dissociation inhibitors (RhoGDIs) also play a key role in regulating the activities of Rho GTPase family proteins [13], as they bind to most GDP-bound Rho GTPases, preventing them from binding to RhoGEFs or their effector molecules [14]. Thus, they are considered negative regulators of Rho GTPases. However, RhoGDIs can also function as chaperons for Rho GTPases, regulating the delivery and extraction of Rho GTPases to their action sites [15]. When Rho GTPases dissociate from RhoGDIs, they can enter the membrane, where they are activated by RhoGEFs. Re-association with RhoGDIs is necessary for the recycling of Rho GTPases [16, 17].

There are three members of the RhoGDI family in mammals, namely, RhoGDI1, RhoGDI2, and RhoGDI3. RhoGDI1 is ubiquitously expressed in all mammalian organs; whereas RhoGDI2 is hematopoietic cell-specific, and RhoGDI3 is expressed in the brain, kidney, and testis [18-20]. Accumulating evidence shows that RhoGDI1 is involved in tumorigenesis and cancer progression. Even though anomalous RhoGDI1 expression has been detected in a variety of human cancers, the exact type of dysregulation is dependent on cancer type [21, 22]. In some human cancers, such as lung and brain cancers, RhoGDI1 is downregulated, and its expression level is actually negatively correlated with the degree of malignancy [23, 24]. In contrast, upregulated expression of RhoGDI1 is observed in hepatocellular carcinoma and has been associated with high metastatic potential [25]. Although, as mentioned above, many studies have provided evidence that suggests the relevance of RhoGDI1 expression in human cancer progression, the mechanism regulating RhoGDI1 expression has been less studied. In the present study, we investigated the mechanism underlying the regulation of RhoGDI1 degradation by CUL3-based ubiquitin ligase complexes.

## Materials and Methods

### DNA Constructs

Full-length human RhoGDI1 cDNA (GenBank Accession: NM_004309), RhoGDI2 cDNA (GenBank Accession: NM_001175), and CUL3 clones were obtained from Origene (USA). A human KCTD5 cDNA clone (GenBank Accession: BC064381) was obtained from GenScript (USA). Human dominant negative CUL3 clones were obtained from addgene (USA). Epitope tags were added to the constructs using synthesized primers. Flag-tagged RhoGDI1, Flag-tagged RhoGDI2, Myc-tagged CUL3, HA-tagged DN-CUL3, and KCTD5 were generated by PCR and inserted into pCDNA3.1.

### Cell Culture and Transfections

HEK293T cells were obtained from the American Type Culture Collection (USA). Cells were cultured at 37°C in a humidified 5% CO_2_ incubator in DMEM (Invitrogen, USA) supplemented with 10% FBS and antibiotics (100 U/ml penicillin and 100 µg/ml streptomycin). For transient transfection, cells were seeded in 6-well plates or 100 mm dishes overnight. The following day, they were transfected with mock, Flag-RhoGDI1, Flag-RhoGDI2, KCTD5, and/or CUL3 (wild-type or truncated dominant negative mutants)-expressing vectors using the XtremGene 9 transfection reagent (MilliporeSigma, USA) following the instructions of the manufacturer. Two days after transfection, cells were harvested and subjected to western blot or immunoprecipitation analysis.

### Western Blot Analysis

Western blot analysis was performed as described previously, with minor modifications [26]. In brief, lysis buffer (20 mM Tris [pH 8.0], 137 mM NaCl, 10% glycerol, 1% Nonidet P-40, 1 mM phenylmethylsulfonyl fluoride, protease inhibitor mixture [Roche, Basel, Switzerland], and 1 mM sodium vanadate) was used to collect protein from the transfected cells. Heat-denatured protein samples were separated by SDS-PAGE and transferred to polyvinylidene difluoride membranes (Amersham Biosciences, UK). Subsequently, the membranes were incubated in TBST supplemented with 5% non-fat dry milk or 5% BSA and probed with the following antibodies: anti-Flag HRP (MilliporeSigma, A8592), anti-HA HRP (Roche, 12013819011), anti-Myc HRP (MilliporeSigma, 16-213), and anti-KCTD5 (Abcam, UK; ab214096).

### Co-Immunoprecipitation Assay

Flag-RhoGDI1- or Flag-RhoGDI2-expressing vectors were co-transfected with Myc-tagged CUL3- or KCTD5-expressing vectors into HEK293T cells in 100 mm plates using the XtremeGene 9 transfection reagent. After 48 h, cells were harvested and lysed using lysis buffer (see “Cell culture and transfections”). Aliquots of cell lysates containing 1 mg protein were immunoprecipitated with 1 mg anti-Flag (MilliporeSigma, F1804) or anti-Myc (MilliporeSigma, 05-724) antibodies at 4°C overnight. The next day, protein A/G PLUS-agarose beads (Santa Cruz Biotechnology, Santa Cruz, California, USA; sc-2025) were added to the samples and incubated at 4°C for 1 h. After extensive washing with lysis buffer (3 times), the beads were spun down and resuspended in 30 μl of 2× SDS sample buffer. After being boiled for 5 min, immunoprecipitates and whole lysates were separated by SDS-PAGE and immunoblotted with the appropriate antibodies as described in the corresponding figure legends.

### RNA Interference Experiments

The KCTD5 siRNA (54442-1) and the AccuTarget Negative Control siRNA (siCon) were obtained from Bioneer (Korea). Transient transfection of each siRNA was accomplished using Lipofectamine RNAiMAX (Invitrogen) according to the instructions of the manufacturer. After incubation for 48 h, cells were harvested, and the efficiency of each siRNA was confirmed by immunoblotting.

### Ubiquitination Assay

HEK293T cells were transfected with HA-ubiquitin, Flag-RhoGDI1, Myc-CUL3, and/or KCTD5 expression vectors. After incubation for 36 h, cells were treated with 10 μM MG132 for 12 h to block proteasomal degradation of the RhoGDI1 protein. Cell lysates were collected and immunoprecipitated with anti-Flag antibody and A/G agarose beads to pull down Flag-RhoGDI1. The immunoprecipitates were subjected to immunoblotting with anti-HA antibody to detect polyubiquitylated RhoGDI1.

### Stability Assay

HEK293T cells transfected with HA-ubiquitin and Flag-RhoGDI1 expression vectors were treated with 20 μg/ml cycloheximide (CHX) to block further protein synthesis. RhoGDI1 levels were determined by western blotting at various times up to 12 h after the addition of CHX.

### Statistical Analysis

Data were obtained from at least three independent experiments. All quantitative data are presented as means ± standard deviations. Differences were assessed using Student’s *t*-tests, with *p* < 0.05 considered statistically significant.

## Results

### CUL3 Interacts with RhoGDI1 and Promotes Its Ubiquitination

**Fig. 1:**
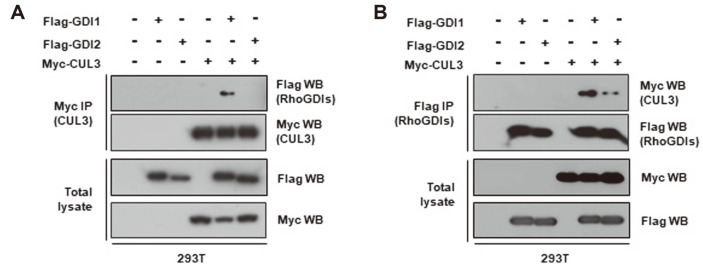
CUL3 interacts with RhoGDI1. HEK293T cells were co-transfected Myc-CUL3 with Flag-RhoGDI1 or Flag-RhoGDI2. (**A**) Cell lysates were immunoprecipitated with anti-Myc antibody. Immunoprecipitates and total lysates were immunoblotted with anti-Myc and anti-Flag antibodies. (**B**) Cell lysates were immunoprecipitated with anti-Flag antibody. Immunoprecipitates and total lysates were immunoblotted with anti-Myc and anti-Flag antibodies.

In our previous study, mass spectrometry revealed the presence of CUL3 in RhoGDI1 immune complexes from HEK293T cells overexpressing the latter [26]. In the current study, to confirm the interaction between these two proteins, HEK293T cells were transfected with Myc-tagged CUL3 and Flag-tagged RhoGDI1 or Flag-tagged RhoGDI2 expression vectors. Cell lysates were immunoprecipitated with an anti-Myc antibody. Western blot analysis detected RhoGDI1 but not RhoGDI2 in the Myc-tagged CUL3 immune complexes (Fig. 1A). Moreover, CUL3 was found in Flag-tagged RhoGDI1 immune complexes (Fig. 1B).

**Fig. 2:**
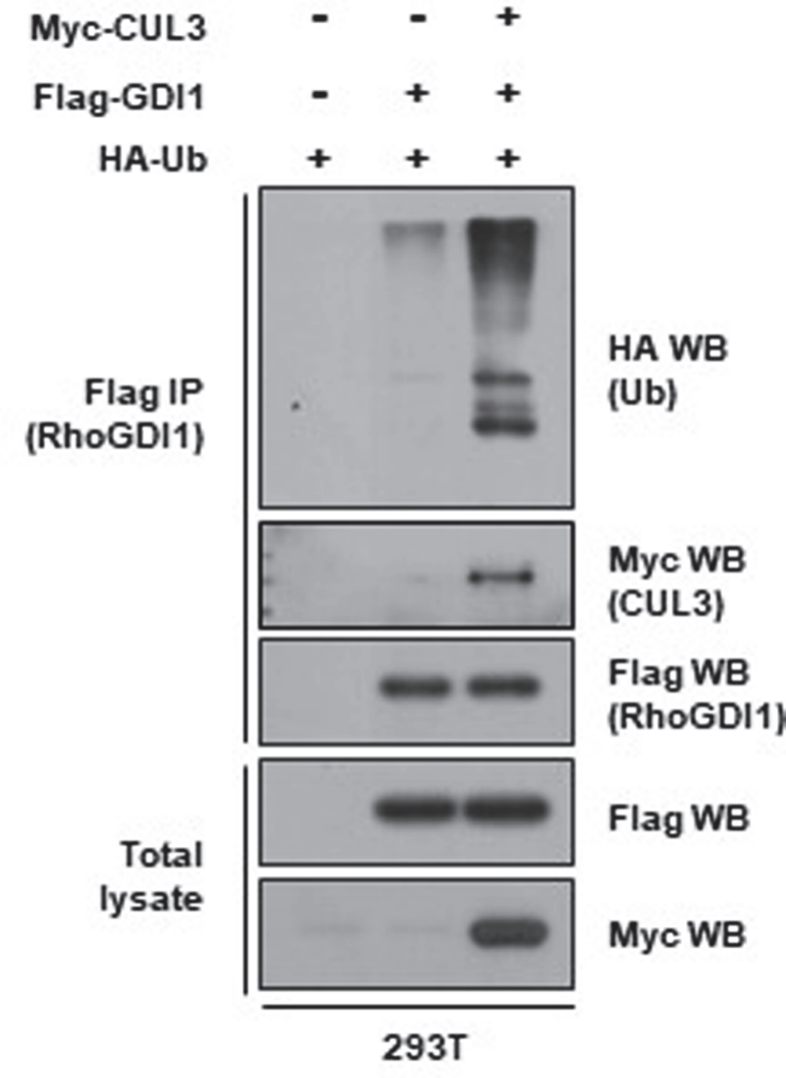
CUL3 overexpression enhances the ubiquitination of RhoGDI1. HEK293T cells were co-transfected with Flag-RhoGDI1, Myc-CUL3, or HA-ubiquitin, and treated with 10 μM of MG132 for 12 h. Cell lysates were immunoprecipitated with anti-Flag antibody. Immunoprecipitates and total lysates were immunoblotted with anti-HA, anti-Flag, or anti-Myc antibodies.

Because CUL3 is a component of Cullin-RING E3 ubiquitin ligase complexes (CRL3), which are involved in the ubiquitination and degradation of target proteins [6], we next examined whether it affected the ubiquitination of RhoGDI1. To this end, HEK293T cells were transfected with Flag-RhoGDI1 and HA-ubiquitin with or without Myc-CUL3. Enhanced conjugation of ubiquitin to RhoGDI1 was detected in CUL3-overexpressing cells compared to levels in mock vector-expressing cells (Fig. 2). Collectively, these results indicate that CRL3 mediates the ubiquitination of RhoGDI1.

### KCTD5 Mediates the Ubiquitination of RhoGDI1 by CUL3

**Fig. 3:**
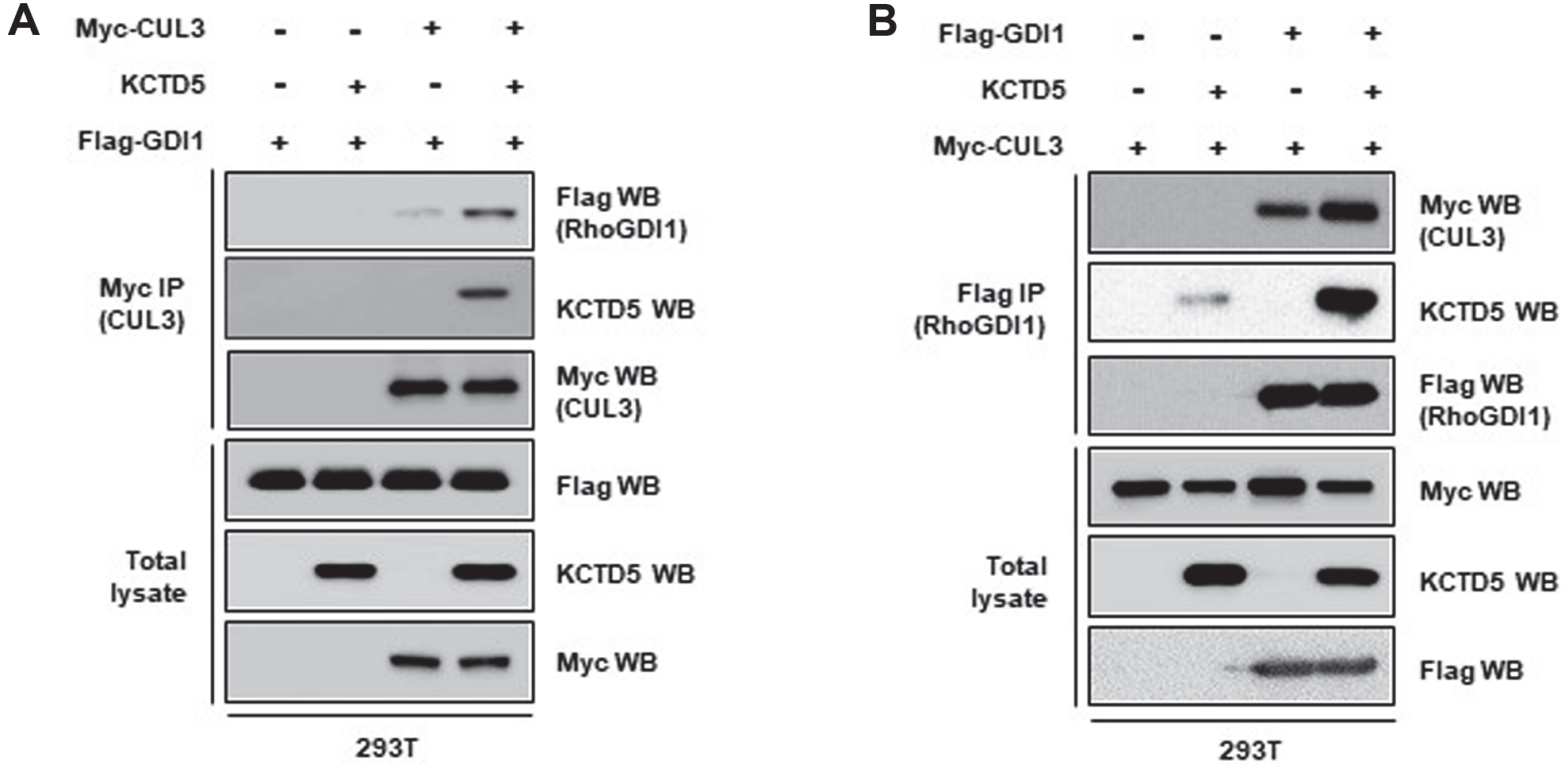
KCTD5 links the interaction of RhoGDI1 with CUL3. HEK293T cells were co-transfected with Flag-RhoGDI1, Myc-CUL3 or KCTD5 as indicated. (**A**) Cell lysates were immunoprecipitated with anti-Myc antibody. Immunoprecipitates and total lysates were immunoblotted with anti-Myc, anti-Flag or anti-KCTD5 antibodies. (**B**) Cell lysates were immunoprecipitated with anti-Flag antibody. Immunoprecipitates and total lysates were immunoblotted with anti-Myc, anti-Flag or anti-KCTD5 antibodies.

It has been reported that KCTD5 is a component of CRL3, which suggests that it may play a role in CRL3-mediated ubiquitination [27-29]. As this protein has also been found in RhoGDI1 immune complexes by mass spectrometric analysis [26], we decided to examine whether KCTD5 links RhoGDI1 to CUL3. To this end, we co-transfected HEK293T cells with various combinations of Flag-RhoGDI1, Myc-CUL3, and KCTD5 expression vectors, and performed immunoprecipitation analysis with anti-myc antibody to capture CUL3 immune complexes. Western blot analysis of these complexes showed that KCTD5 is associated with RhoGDI1 (Fig. 3A). More importantly, even though RhoGDI1 could be detected in CUL3 immune complexes in the absence of co-transfection with the KCTD5 expression construct, the amount of immunoprecipitated RhoGDI1 was impressively higher when KCTD5 was overexpressed (Fig. 3A). Similarly, when immunoprecipitation was performed with anti-FLAG antibody to capture RhoGDI1 immune complexes, the amount of precipitated CUL3 markedly increased when KCTD5 was also overexpressed (Fig. 3B). These data suggest that a tri-molecular complex is formed where RhoGDI1 interacts with KCTD5, which binds to CUL3.

**Fig. 4:**
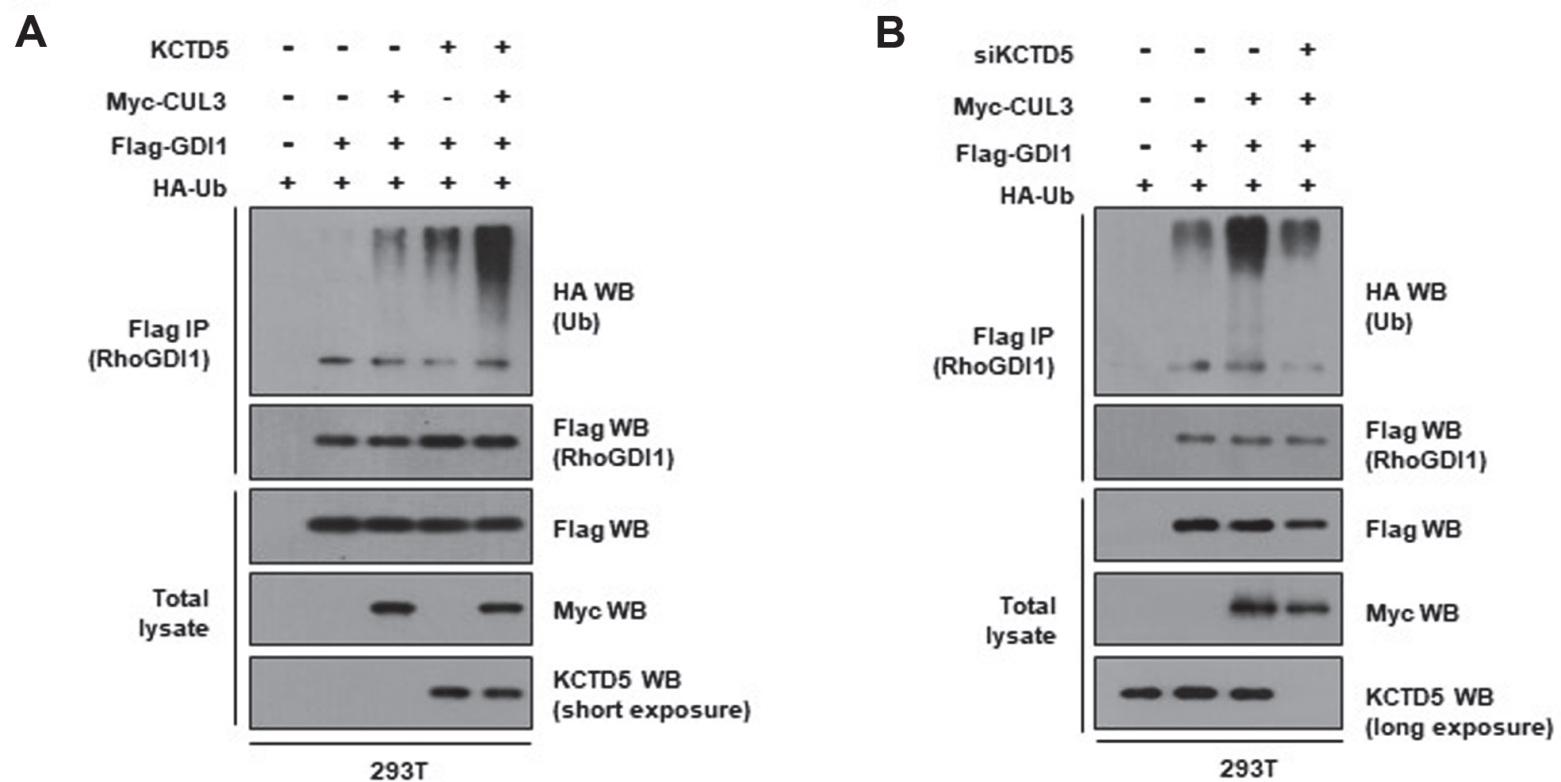
CUL3/KCTD5 mediates the ubiquitination of RhoGDI1. (**A**) HEK293T cells were co-transfected with Flag-RhoGDI1, Myc-CUL3, KCTD5 or HA-ubiquitin as indicated, and treated with 10 μM of MG132 for 12 h. Cell lysates were immunoprecipitated with anti-Flag antibody. Immunoprecipitates and total lysates were immunoblotted with anti-HA, anti-Myc, anti-Flag or anti-KCTD5 antibodies. (**B**) HEK293T cells were transfected with the control or KCTD5 siRNA. After 18 h, cells were co-transfected with Flag-RhoGDI1, Myc-CUL3 or HA-ubiquitin as indicated. 36 h after transfection, cells were treated with 10 μM of MG132 for 12 h. Cell lysates were immunoprecipitated with anti-Flag antibody. Immunoprecipitates and total lysates were immunoblotted with anti-HA, anti-Myc, anti-Flag or anti-KCTD5 antibodies.

Next, we examined whether KCTD5 affects the ubiquitination of RhoGDI1 by CUL3. To this end, we cotransfected HEK293T cells with various combinations of Flag-RhoGDI1, HA-ubiquitin, Myc-CUL3, and KCTD5 expression vectors, and then performed immunoprecipitation with anti-Flag antibody. Western blot analysis of the RhoGDI1 immune complexes using anti-HA antibody showed that the conjugation of ubiquitin to RhoGDI1 is most robust when both KCTD5 and CUL3 were overexpressed (Fig. 4A). In contrast, KCTD5 depletion by siRNA significantly attenuated the ubiquitination of RhoGDI1 (Fig. 4B). As a whole, these findings strongly suggest that KCTD5 is necessary for CUL3-mediated RhoGDI1 ubiquitination.

### CUL3/KCTD5 Downregulates RhoGDI1 Stability

**Fig. 5:**
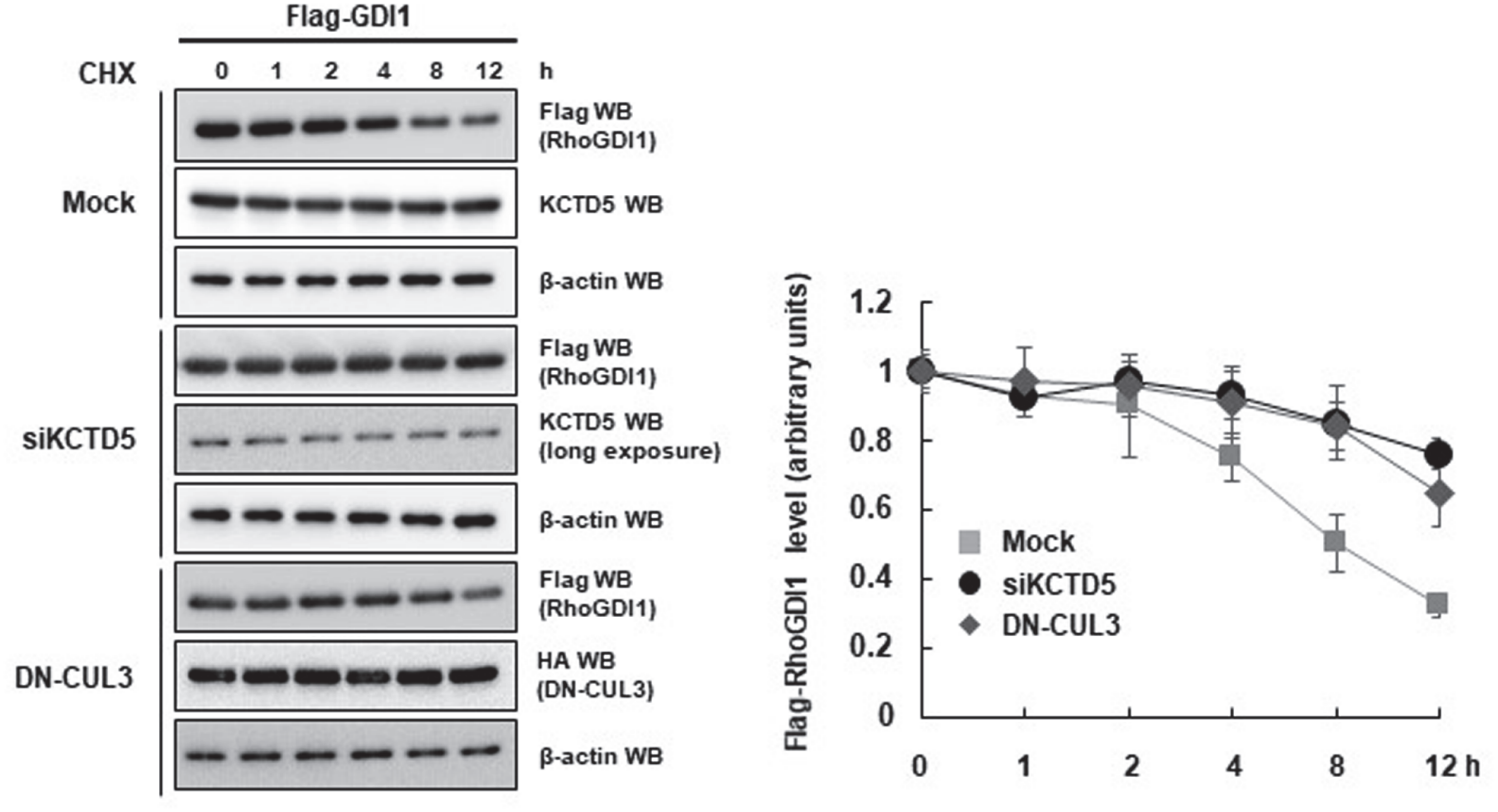
CUL3/KCTD5 downregulates the stability of RhoGDI1 protein. Flag-RhoGDI1 was co-transfected into HEK293T cells with KCTD5 siRNA or DN-CUL3. Cells were treated with 20 μg/ml CHX for indicated times. Cell lysates were immunoblotted with anti-Flag antibody. The right panel presents the quantification of the data from three independent experiments.

Based on the collaboration of CUL3 and KCTD5 in RhoGDI1 ubiquitination, we hypothesized that these proteins might interact within a ubiquitin ligase complex that marks RhoGDI1 for degradation. To determine the effect of CUL3 and KCTD5 on RhoGDI1 stability, we monitored the levels of the latter in CHX-treated HEK293T cells overexpressing HA-ubiquitin and Flag-RhoGDI1. As seen in Fig. 5 (left panel), the abundance of RhoGDI1 was reduced by half within 8 h of CHX treatment. In contrast, depletion of KCTD5 by siRNA significantly reduced the degradation of RhoGDI1 protein (Fig. 5, right panel). The extent of the reduction in RhoGDI1 degradation was similar to that observed when a truncated dominant negative form of CUL3 (DN-CUL3) was overexpressed (Fig. 5, right panel). These results clearly suggest that CUL3/KCTD5 mediates the ubiquitination-mediated degradation of RhoGDI1.

## Discussion

RhoGDI1 is involved in a wide range of cellular processes, including cell adhesion, migration, and proliferation [16]. It plays important roles in regulating the activities of Rho GTPase family members, such as RhoA, Rac1, and Cdc42. Altered expression of RhoGDI1 is observed in many human cancers and is associated with cancer progression and chemoresistance [22]. A recent study reported that activation transcription factor 4 (ATF4) modulates Rho GTPase protein abundance and function by transcriptionally regulating RhoGDI1 expression, thereby promoting cell motility in hippocampal and cortical neurons [30]. RhoGDI1 expression is also post-transcriptionally regulated by microRNAs such as miR-25, which promotes cell proliferation and migration by downregulating RhoGDI1 expression in hepatocellular carcinoma [31], and miR-151, which is expressed together with focal adhesion kinase (FAK) and promotes the invasion and metastasis of hepatocellular carcinoma cells by inhibiting RhoGDI1 expression [32].

In this study, we report a novel molecular mechanism for the post-translational regulation of RhoGDI1 expression that is dependent on CUL3-mediated ubiquitination. KCTD5 seems to also be an important part of this mechanism. Its overexpression enhanced both the interaction of RhoGDI1 with CUL3 (Fig. 3), suggesting that it functions as an adaptor protein for interaction between these two proteins, as well as CUL3-mediated RhoGDI1 ubiquitination. In contrast, its depletion by siRNA attenuated the ubiquitination of RhoGDI1 (Fig. 4).

Both KCTD5 depletion and DN-CUL3 overexpression increased the stability of RhoGDI1 protein. However, the inhibition of RhoGDI1 degradation by either treatment was incomplete, suggesting that there may be other mechanisms for RhoGDI1 degradation. In fact, a recent study reported that FBW E3 ubiquitin ligase can interact with and degrade RhoGDI1 in a proteasomal-dependent manner [33]. In addition, angiotensin II was found to regulate the stability of RhoGDI1 by sumoylation and ubiquitination, thus affecting vascular smooth muscle cell proliferation [34].

Ubiquitination is the post-translational modification process by which ubiquitin is attached to a lysine residue in the substrate. Ubiqutination controls the localization, activity, and stability of Rho GTPases, including RhoA, RhoB and Rac1 [35, 36]. For example, SMAD ubiquitination regulatory factor 1 (Smurf1) promotes the ubiquitination of active GTP-RhoA, leading to its degradation at the specific cellular protrusion of migrating cells [37]. On the other hand, CUL3/BACURD catalyzes the ubiquitination of inactive GDP-RhoA [38]. Moreover, the CUL3-Rbx1-KCTD10 complex controls the endothelial barrier function via the ubiquitination of RhoB [39]. Our current study reveals that the CUL3/KCTD5 complex promotes the ubiquitination of RhoGDI1 and modulates its stability. Deregulation of the Rho GTPase pathway is implicated in various diseases, such as neurodegenerative and developmental disorders, as well as in tumorigenesis and cancer progression [10]. RhoGDI1 is also associated with cancer cell migration, invasion, metastasis, and chemoresistance via regulation of Rho GTPase activity [22]. Therefore, our future efforts will focus on verifying the effect of the CUL3/KCTD5-mediated regulation of RhoGDI1 stability on cancer progression.

In conclusion, we identified CUL3 and KCTD5 as novel binding proteins for RhoGDI1. The CUL3/KCTD5 complex controls the stability of RhoGDI1 by promoting its ubiquitination. Our results provide mechanistic insight into the regulation of RhoGDI1 degradation by CRL3 complexes.
